# Genomes of the ex-type strains of *Elsinoë mangiferae* and *E. perseae*, the causal agents of scab on mango and avocado

**DOI:** 10.1093/g3journal/jkag177

**Published:** 2026-07-02

**Authors:** Priscila Chaverri, Efraín Escudero-Leyva, Pepijn W Kooij, Cambly Morales-Lemus, Catalina Salgado-Salazar

**Affiliations:** Department of Natural Sciences, Bowie State University, 14000 Jericho Park Road, Bowie, MD 20715, United States; Centro de Investigaciones en Productos Naturales (CIPRONA), Universidad de Costa Rica, San Pedro, San José 11501, Costa Rica; Centro Nacional de Innovaciones Biotecnológicas (CENIBiot, CeNAT, CONARE), Pavas, San José 10109, Costa Rica; Centro de Investigaciones en Productos Naturales (CIPRONA), Universidad de Costa Rica, San Pedro, San José 11501, Costa Rica; Estación Experimental Agrícola Fabio Baudrit, Escuela de Agronomía, Universidad de Costa Rica, Alajuela 20113, Costa Rica; Department of General and Applied Biology, São Paulo State University (UNESP), Institute of Biosciences, Rio Claro, São Paulo 13506-900, Brazil; Department of Natural Sciences, Bowie State University, 14000 Jericho Park Road, Bowie, MD 20715, United States; Mycology and Nematology Genetic Diversity and Biology Laboratory, U.S. Department of Agriculture, Agriculture Research Service (USDA-ARS), 10300 Baltimore Avenue, Beltsville, MD 20705, United States

**Keywords:** ACT-toxin II, Dothideomycetes, elsinochrome, hemibiotroph, hybrid genome assembly, Myringiales, necrotroph

## Abstract

*Elsinoë* species are slow-growing, hemibiotrophic to necrotrophic fungi that cause scab diseases on economically important fruit crops. Genome resources for many host-specific species remain limited. We report high-quality draft genome assemblies for the ex-type strains of *Elsinoë mangiferae* (CBS 226.50) and *E. perseae* (CBS 406.34), causal agents of mango and avocado scab, respectively. Among 5 approaches tested, a Nanopore-only NextDenovo assembly produced the most contiguous genomes, yielding 24.5 Mb (*E. mangiferae*) and 25.1 Mb (*E. perseae*) assemblies with 13 and 18 contigs, respectively, BUSCO completeness scores of ∼94%, and multiple putative telomere-to-telomere chromosomes. Gene prediction identified 9,134 and 9,243 genes, respectively. Functional annotation revealed enrichment of metabolic and regulatory pathways, including those involved in posttranslational modification, protein transport, and secondary metabolism. Carbohydrate-active enzyme repertoires were small but conserved, consistent with stealth pathogenicity strategies and low plant cell wall degradation. Both genomes encoded large secretomes (>850 proteins), diverse protease repertoires (>300 proteins), Ecp2-like effector proteins, and multiple biosynthetic gene clusters, including clusters with similarity to those associated with elsinochrome and ACT-toxin II biosynthesis, some of which may contribute to host-pathogen interactions and disease development. A large fraction of genes lacked functional characterization, suggesting incomplete databases and/or the presence of lineage-specific genes potentially involved in virulence or host adaptation. These genome resources fill critical gaps for underrepresented *Elsinoë* species and provide taxonomically anchored references essential for diagnostics, comparative genomics, and research into the molecular basis of host specificity and pathogenicity in scab-causing fungi.

## Introduction

*Elsinoë* (*Elsinoaceae, Myringiales, Dothideomycetes, Ascomycota*) species are hemibiotrophic to necrotrophic, slow-growing, and highly host-specific fungi that infect a range of economically important fruit crops, including citrus, avocado, and mango (Jenkins 1934; Bitancourt and Jenkins 1946; Fan et al. 2017; Belizaire et al. 2024; Pham et al. 2025). The disease they cause, commonly referred to as “scab,” is characterized by corky, rough lesions on infected plant tissues, often covering extensive areas of the fruit surface. Although primarily cosmetic, this damage can severely impact the visual quality of the fruit, reducing its marketability and export value (Marais 2004; Ramírez-Gil et al. 2019). International trade of susceptible fruits is frequently regulated due to the restricted distribution or absence of *Elsinoë* spp. in some regions and the associated risk of pathogen introduction. Notably, *E. australis* (sweet orange scab) and *E. fawcettii* (citrus scab), both of which infect *Citrus* spp., are quarantine-regulated pathogens in several regions, including the European Union, the United States, and Australia (EFSA Panel on Plant Health (PLH) et al. 2017; USDA-APHIS 2025).

Due to the sparsity of distinguishing morphological features, molecular methods are typically required for definitive diagnosis (Fan et al. 2017; Belizaire et al. 2024). GenBank currently has 25 genome assemblies representing nine *Elsinoë* species; most of these genomes belong to *E. australis* and *E. fawcettii*. However, other noteworthy and globally distributed *Elsinoë* species, e.g. *E. mangiferae* and *E. perseae*, which cause scab on mango and avocado, respectively, remain underrepresented in genome databases (Marais 2004; Gupta et al. 2020; Pham et al. 2025). These species affect widely traded tropical fruits and have considerable phytosanitary relevance. For example, *E. perseae* has been intercepted at US ports of entry an average of 167 times per year since 1997 and is currently listed as a quarantine pest in Hawaii (USDA Agricultural Risk Management, ARM, database; accessed by M.K. Romberg, July 11, 2025, pers. comm.). *Elsinoë mangiferae* has been intercepted an average of 172 times per year since 1984 but is not classified as a quarantine organism in any US state or territory (USDA-ARM database). Although 1 genome assembly for *E. perseae* is available (Gañán-Betancur and Gazis 2023), it was not generated from the ex-type strain. To date, no genome has been sequenced for *E. mangiferae*, stressing the need to generate high-quality, taxonomically anchored genomic resources for both species.

The objective of this study is to report the genomes of the ex-type strains of *Elsinoë mangiferae* (CBS 226.50) and *E. perseae* (CBS 406.34). These 2 species were originally described from specimens collected in Cuba (Bitancourt and Jenkins 1946) and Florida, United States (Jenkins 1934), respectively, and cultures derived from the original specimens were designated as ex-types (Fan et al. 2017). Sequencing genomes from types is crucial for establishing a taxonomically accurate genomic baseline. Because these strains are directly linked to the original species descriptions and names, they provide an unambiguous reference for species identification. This is particularly important in *Elsinoë*, a genus with cryptic species and overlapping morphological features, where the use of non-type strains can result in misidentification or taxonomic confusion (Fan et al. 2017; Belizaire et al. 2024). Moreover, genome sequences from ex-type strains are foundational for developing reliable molecular diagnostic tools, such as species-specific PCR primers, qPCR assays, and metabarcoding markers. Finally, the inclusion of type-derived genomes in public repositories enhances the quality and reliability of genomic databases by enabling the distinction between correctly identified species and misidentified or poorly annotated genomes, especially from environmental or non-type samples/specimens.

## Materials and methods

### Fungal cultures and DNA extraction

Ex-type strains of *Elsinoë mangiferae* (CBS 226.50) and *E. perseae* (CBS 406.34) were obtained from the Westerdijk Fungal Biodiversity Institute (CBS-KNAW, Utrecht, The Netherlands). The fungal cultures were reactivated following CBS-KNAW's instructions, followed by inoculation into 250 mL flasks containing Millipore malt-extract-broth (2% MEB). These were then grown and incubated for ca. 7 d at 21 °C in a MaxQ 4000 Benchtop Orbital Shaker (ThermoFisher Scientific, Waltham, Massachusetts, United States) at 24 °C and 110 rpm. Fresh mycelium was harvested using a Buchner funnel system, sterile filter paper, and vacuum, then dried with sterile paper towels, immediately flash-frozen with liquid nitrogen, and stored at −80 °C for a maximum of 5 d. Approximately 100 mg of frozen mycelia were then ground using a mortar and pestle with liquid nitrogen throughout the process. Each sample was ground separately at different times to avoid cross-contamination. DNA was extracted from the ground mycelia with the OmniPrep Genomic DNA Isolation kit (G-Biosciences, St. Louis, Missouri, United States). The extracted DNA was then cleaned with Zymo Genomic DNA Clean & Concentrator (Zymo Research, California, United States). Initial purity and quantity of genomic DNA were assessed with a DS-11 FX Spectrophotometer and Fluorometer (DeNovix Inc., Wilmington, Delaware, United States) and then after pooling or dilution using a Qubit (ThermoFisher Scientific, Waltham, Massachusetts, United States) dsDNA BR assay kit.

### Genome sequencing

To attempt to produce a high-quality genome assembly, genomic DNA was sequenced using both Illumina and Oxford Nanopore (ONT) technologies. Illumina libraries were prepared using the TruSeq DNA Nano kit (Illumina, San Diego, California, United States) and quantified using a D1000 ScreenTape on a TapeStation System 4150 (Agilent, Santa Clara, California, United States). Libraries were sequenced on an Illumina MiSeq as 2 × 300 paired-end reads using a MiSeq version 3.0, 600 cycle reagent kit. Before ONT library preparation, high-molecular-weight genomic DNA quality was assessed using a Genomic DNA ScreenTape assay on a TapeStation System (Agilent Technologies, Santa Clara, California, United States), and both the DNA Integrity Number (DIN) and fragment size distribution were evaluated to ensure suitability for long-read sequencing. ONT libraries were then prepared using the Ligation Sequencing gDNA kit (SQK-LSK110) (Oxford Nanopore Technologies, Oxford, U.K.), and 12 µL of the final libraries were loaded onto an R9.4.1 flow cell and sequenced using a MinION Mk1C device (Oxford Nanopore Technologies).

### Genome assembly and evaluation

Five assembly approaches were used: 1 de novo hybrid (MaSuRCa) for Illumina and ONT reads, and 4 for ONT-only (Flye, Hifiasm, SmartDenovo, and NextDenovo). QUAST v5.2.0 (Gurevich et al. 2013) was used to evaluate assembly quality and identify the most suitable for downstream analyses. All bioinformatic analyses were performed on the University of Costa Rica high-performance computing cluster (UCR-HPC). The pipeline used is publicly available in GitHub (https://github.com/EEscuderoL/Elsinoe-genomes-pipeline). This Whole Genome Shotgun (WGS) project has been deposited at DDBJ/ENA/GenBank under the accessions JBPVOW000000000 and JBPVOX000000000 (BioProject PRJNA1295512).

For the hybrid assembly, Illumina raw reads were assessed for quality using FastQC v.12.1 (Andrews 2010) and trimmed with Fastp v.0.23.3 (Chen et al. 2018). For the long reads derived from ONT, trimming was done with Porechop v.0.2.4 (Bonenfant et al. 2023). Then, a *de novo* hybrid assembly was performed with MaSuRCA v.4.1.1 (Zimin et al. 2013), using default settings and specifying the directories for each read type. Finally, the assembly was polished with NextPolish v.1.4.1 (Hu et al. 2020).

For the ONT-only assemblies, long reads were adapter-trimmed as previously described using Porechop. Genome assemblies were generated independently using 4 long-read assemblers: (1) Flye v2.9.5-b1801 (Kolmogorov et al. 2019) with the parameters –read-error 0.05 and –genome-size 50 m; (2) Hifiasm v0.25.0-r726 (Cheng et al. 2021) with –primary and –ont options to generate primary assemblies from ONT reads; (3) SmartDenovo v1.0.0 (Liu et al. 2021) with default parameters; and (4) NextDenovo v2.5.2 (Hu et al. 2024) using a configuration file with the following settings: read_cutoff = 1k, genome_size = 50 m, sort_options = -m 20 g -t 8, pa_correction = 4, and correction options -p 8.

To assess chromosome completeness, the first and last 10 kb of each contig were extracted using Seqkit v.2.10.0 (Shen et al. 2016). The extracted terminal regions were screened for the canonical fungal telomeric repeat motif (TTAGGG/CCCTAA) (Podlevsky et al. 2008) using regular expression searches implemented with grep. Contigs containing telomeric repeats at both ends were considered putative telomere-to-telomere (T2T) assemblies, whereas contigs containing a telomeric repeat at only 1 end were interpreted as extending to a single chromosome terminus.

### Gene prediction and functional annotation

The genome assemblies were converted to FASTA format using Seqkit. Genome completeness was evaluated with BUSCO v.6.0.0 (Waterhouse et al. 2018) using the “dothideomycetes_odb10” database as the reference. Gene prediction and annotation were performed using the Funannotate v.1.8.17 pipeline (Palmer and Stajich 2020), which integrates *ab initio* predictors including Augustus v.3.5.0 (Stanke et al. 2006; Hoff and Stanke 2019). Augustus was run using the *Elsinoë* (=*Sphaceloma*) *murrayae* species model as the training set. Funannotate annotates predicted proteins using multiple databases and tools, including InterPro for protein domains and families, dbCAN3 for carbohydrate-active enzymes (CAZymes), MEROPS for proteases, and SignalP for the prediction of secreted proteins based on the presence of signal peptides (Palmer and Stajich 2020). EggNOG-mapper v2.1.12 (Cantalapiedra et al. 2021) was used to identify orthologous groups and KEGG pathways, with protein alignments performed using the DIAMOND algorithm (Buchfink et al. 2021). Lastly, the fungal version of antiSMASH v.8.0 (Blin et al. 2025) was used to identify biosynthetic gene clusters using the previously obtained gff3 and gbk files. Gene prediction and annotation files are available in Figshare (https://doi.org/10.6084/m9.figshare.31855519).

### Phylogenomic species tree

To evaluate the phylogenetic position of our 2 strains, 25 additional *Elsinoë* genomes were retrieved from GenBank (Supplementary Table 1). *Cercospora beticola* (*Mycosphaerellaceae, Capnodiales, Dothideomycetes*) was used as the outgroup. After obtaining the protein files with Augustus, OrthoFinder v.3.0.1b1 (Emms and Kelly 2019) was used to find single-copy orthologous genes, selecting BLAST as the search tool. The resulting concatenated alignment was analyzed with IQ-TREE2 (Minh et al. 2020) to infer a phylogeny, with ultrafast bootstrap analysis set to a maximum of 1,000 replicates and automatically stopping upon convergence.

## Results and discussion

### Genome assemblies, gene prediction, and comparison with other *Elsinoë* genomes

After reads were quality filtered, a total of 10.6 million paired-end sequencing reads (Illumina) and 1.8 million ONT reads were obtained for *E. mangiferae*, and 10.6 million paired-end sequencing reads (Illumina) and 4.5 million ONT reads were obtained for *E. perseae*. Comparison of the 5 assembly approaches indicated that NextDenovo produced the most contiguous and complete assemblies (Table 1). Therefore, the NextDenovo assemblies were selected for all downstream analyses. The *E. mangiferae* assembly comprised 13 contigs, with a total genome size of 24.5 Mb, largest contig ∼3.9 Mb, N50 of 2,181,161 bp, and GC content of 52.2% (Table 1). In contrast, *E. perseae* had a slightly larger genome of 25.1 Mb distributed across 18 contigs, largest contig ∼3.3 Mb, N50 of 1,888,356 bp, and GC content of 52.4%. Sequencing depth was 600× and 504× for *E. mangiferae* and *E. perseae*, respectively. Gene prediction identified 9,134 genes (9,060 CDS/mRNA and 74 tRNA) in *E. mangiferae* and 9,243 genes (9,165 CDS/mRNA and 78 tRNA) in *E. perseae* (Table 1, Supplementary Tables 2 and 3). BUSCO completeness was adequate and typical for both genomes, with 3,562 complete BUSCOs (94.1%) in *E. mangiferae* and 3,566 (94.2%) in *E. perseae*. The majority were single-copy and complete BUSCOs, indicating high assembly quality and completeness (Waterhouse et al. 2018; Manni et al. 2021).

**Table 1.** Genome assembly statistics for *Elsinoë mangiferae* CBS 226.50 and *E. perseae* CBS 406.34, and comparison of 5 different assembly approaches.

E. mangiferae CBS 226.50					
Statistic/Assembler	hybrid	masked	flye	smart	nextdenovo
# Contigs	61	71	36	1,948,665	13
Largest contig (bp)	1,536,973	3,799,506	3,829,891	186,572	3,861,580
Total length (bp)	22,813,557	24,211,224	24,193,041	12,720,076,223	24,542,706
GC (%)	52.68	51.65	52.21	51.36	52.2
N50	650,399	1,753,388	1,736,164	22,337	2,181,161
L50	13	5	5	174,156	5
Coverage depth (×)	672	666	566	0	600
# genes^a^	–	–	–	–	9,134
#CDS/mRNA^a^	–	–	–	–	9,060
#tRNA^a^	–	–	–	–	74
Complete BUSCOs^b^	–	–	–	–	3,562 (94.1%)
Complete and single-copy BUSCOs^b^	–	–	–	–	3562
Complete and duplicated BUSCOs^b^	–	–	–	–	0
Fragmented BUSCOs^b^	–	–	–	–	9
Missing BUSCOs^b^	–	–	–	–	215
Total BUSCO groups searched^b^	–	–	–	–	3,786

*E. perseae* CBS 406.34					
Statistic/Assembler	hybrid	masked	flye	smart	nextdenovo
# Contigs	82	144	41	3,060,637	18
Largest contig (bp)	1,266,624	4,321,342	3,054,884	150,846	3,314,964
Total length (bp)	21,733,659	26,101,844	25,011,125	10,546,657,338	25,152,041
GC (%)	52.55	51.5	52.5	50.9	52.4
N50	384,819	1,717,862	1,881,634	8,143	1,888,356
L50	15	5	5	327,377	5
Coverage depth (×)	533	581	477	0	504
# genes^a^	–	–	–	–	9,243
#CDS/mRNA^a^	–	–	–	–	9,165
#tRNA^a^	–	–	–	–	78
Complete BUSCOs^b^	–	–	–	–	3,566 (94.2%)
Complete and single-copy BUSCOs^b^	–	–	–	–	3,562
Complete and duplicated BUSCOs^b^	–	–	–	–	4
Fragmented BUSCOs^b^	–	–	–	–	10
Missing BUSCOs^b^	–	–	–	–	210
Total BUSCO groups searched^b^	–	–	–	–	3,786

^a^Predicted by Funnanotate.

^b^BUSCO completeness against dothideomycetes_odb10 database.

In the context of available *Elsinoë* genomes in GenBank, the assemblies of *E. mangiferae* CBS 226.50 and *E. perseae* CBS 406.34 generated in this study represent 2 of the most complete and deeply sequenced genomes currently available for the genus (Table 2). With only 13 and 18 contigs, respectively, both genomes rank among the most contiguous assemblies, equaled or surpassed only by a few genomes such as *E. ampelina, E. arachidis,* and *E. batatas.* Their high N50 values (∼2.2 Mb for *E. mangiferae* and ∼1.9 Mb for *E. perseae*) further support the quality and continuity of the assemblies. Sequencing depth was exceptionally high, with coverage 500–600× for both species, substantially greater than the typical 100–250× observed across most other *Elsinoë* genomes, enhancing the reliability of structural assembly and gene prediction. In addition, the newly sequenced *E. perseae* genome improves upon a previous draft of another strain (GCA_029448695.1), reducing the number of contigs and providing gene and functional annotations that were previously absent. *Elsinoë mangiferae* CBS 226.50 is the first genome sequenced for this species, filling a critical gap in genomic resources for scab pathogens. Overall, the genome size, GC content, and predicted gene numbers are consistent with those of other species in the genus (Table 2), although both newly assembled genomes are on the lower end of the size and gene-content spectrum. Lastly, telomeric repeats were detected at both ends of several contigs in both genomes (Supplementary Table 4). In *E. mangiferae*, telomeric repeats were detected at both ends of 4 contigs and at 1 end of 7 additional contigs. In *E. perseae*, 3 contigs contained telomeric repeats at both ends, and 2 additional contigs contained a telomeric repeat at a single end. These results suggest that several contigs likely represent complete chromosome-scale assemblies and provide further evidence of the genome assembly's high contiguity.

**Table 2.** Comparison of genome assemblies available in GenBank.

Assembly/Accession	Species	Total Length (bp)	# Contigs	Contig N50	Contig L50	GC %	Coverage (×)	# Genes^a^
GCA_005959805.1	*Elsinoë ampelina*	28,296,786	13	2,703,794	5	49.5	253	NA
GCA_010093995.1	*Elsinoë ampelina*	28,270,677	548	135,044	60	49.5	100	10,233
GCA_013372555.1	*Elsinoë arachidis*	33,184,353	16	3,376,838	5	48	193	NA
GCA_007556505.1	*Elsinoë australis*	23,793,094	25	1,520,745	7	52.5	128	NA
GCA_016625675.1	*Elsinoë australis*	23,866,517	61	947,746	9	52.5	241	NA
GCA_003013795.1	*Elsinoë australis*	23,337,424	453	897,805	9	52.5	245	9,250
GCA_004761985.1	*Elsinoë australis*	26,279,129	1,548	178,957	45	51	100	NA
GCA_004761955.1	*Elsinoë australis*	26,289,625	1,427	174,403	45	51	100	NA
GCA_004761995.1	*Elsinoë australis*	27,122,845	1,626	145,460	54	51	100	NA
GCA_005382405.1	*Elsinoë australis*	26,626,433	1,483	144,636	54	51	160	9,663
GCA_005382375.1	*Elsinoë australis*	26,617,985	1,453	133,418	58	51	160	NA
GCA_005382415.1	*Elsinoë australis*	26,621,968	1,593	131,839	55	51	160	NA
GCA_005382395.1	*Elsinoë australis*	26,604,401	1,425	119,048	65	51	160	NA
GCA_004764485.1	*Elsinoë australis*	24,982,974	3,002	29,780	255	52	100	NA
GCA_017309325.2	*Elsinoë batatas*	26,493,939	13	2,546,814	5	51	168	9,403
GCA_007556565.1	*Elsinoë fawcettii*	26,325,864	109	843,756	11	52.5	128	NA
GCA_007556535.1	*Elsinoë fawcettii*	26,655,928	1,276	952,195	11	53	128	NA
GCA_012977835.1	*Elsinoë fawcettii*	26,011,141	286	694,004	12	52.5	193	10,170
GCA_016625505.1	*Elsinoë fawcettii*	26,483,858	475	668,319	13	52.5	218	NA
GCA_016625515.1	*Elsinoë fawcettii*	25,855,480	474	646,472	15	52.5	235	NA
GCA_016625525.1	*Elsinoë fawcettii*	25,873,468	473	608,276	15	52.5	237	NA
**JBPVOW000000000**	***Elsinoë mangiferae* CBS 226.50**	**24,542,706**	**13**	**2,181,161**	**5**	**52**.**2**	**600**	**9,134**
GCA_002895985.1	*Elsinoë murrayae*	20,718,688	156	357,468	21	54.5	241	8,286
GCA_033846785.1	*Elsinoë necatrix*	25,549,386	49	1,676,154	6	50.5	281	NA
GCA_032393115.1	*Elsinoë necatrix*	23,987,626	68	733,562	10	51.5	102	NA
GCA_029448695.1	*Elsinoë perseae*	23,492,853	169	561,242	14	52.5	84	NA
**JBPVOX000000000**	***Elsinoë perseae* CBS 406.34**	**25,152,041**	**18**	**1,888,356**	**5**	**52**.**4**	**504**	**9,243**

Assemblies for strains in bold were produced for this study. All other information was obtained from GenBank.

^a^NA: not available or not reported by the submitters.

### Phylogenomic tree

**Fig. 1.**
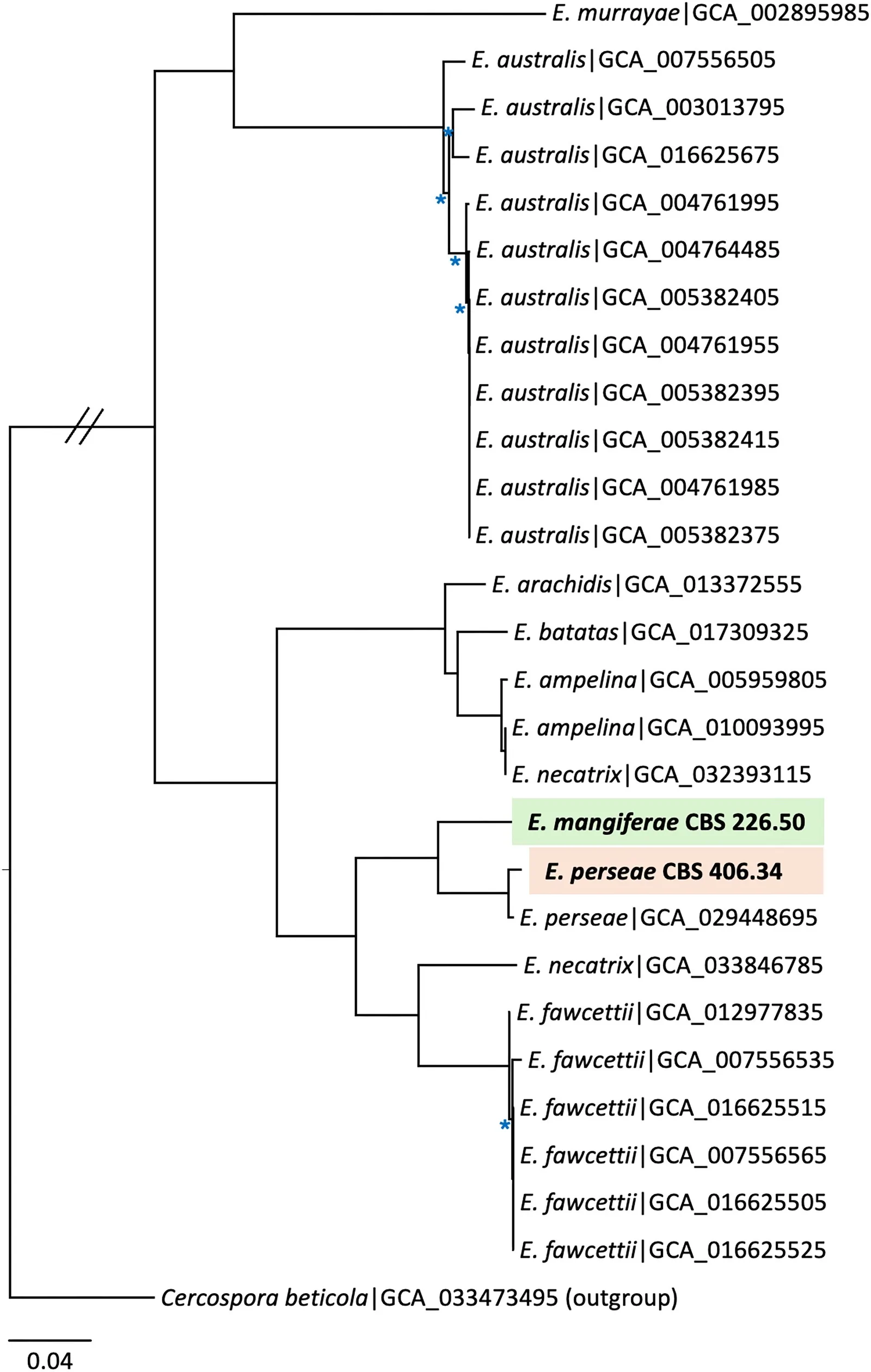
Phylogenomic best species tree of *Elsinoë* spp. produced with OrthoFinder using available genomes. The scale bar represents the number of average amino acid substitutions per aligned site. Nodes with a blue asterisk are supported by bootstrap values less than 70%. Species in bold and highlighted are those sequenced in this study.

OrthoFinder resulted in 11,629 orthogroups (OGs), 1,285 OGs with all species present, and 928 single-copy OGs. OrthoFinder produced an alignment with 1,918,070 columns; 522,969 distinct patterns; 561,038 parsimony-informative; 465,613 singleton sites; and 891,419 constant sites. Although the analysis was set to run for up to 1,000 bootstrap iterations, IQ-TREE terminated after 101 iterations upon reaching convergence. In Fig. 1, *E. mangiferae* and the 2 *E. perseae* strains form a monophyletic group. This is consistent with other previously published multigene phylogenies (Fan et al. 2017; Pham et al. 2025).

### Functional annotations

Funannotate annotated 8,210 and 8,329 proteins using EggNOG; 7,069 and 7,169 using InterPro; 849 and 860 as secreted proteins with SignalP; 411 and 443 as CAZymes with dbCAN; and 301 and 307 as proteases with MEROPS in *E. mangiferae* and *E. perseae*, respectively (Supplementary Tables 5 and 6).

#### COG-based functional annotation

**Fig. 2.**
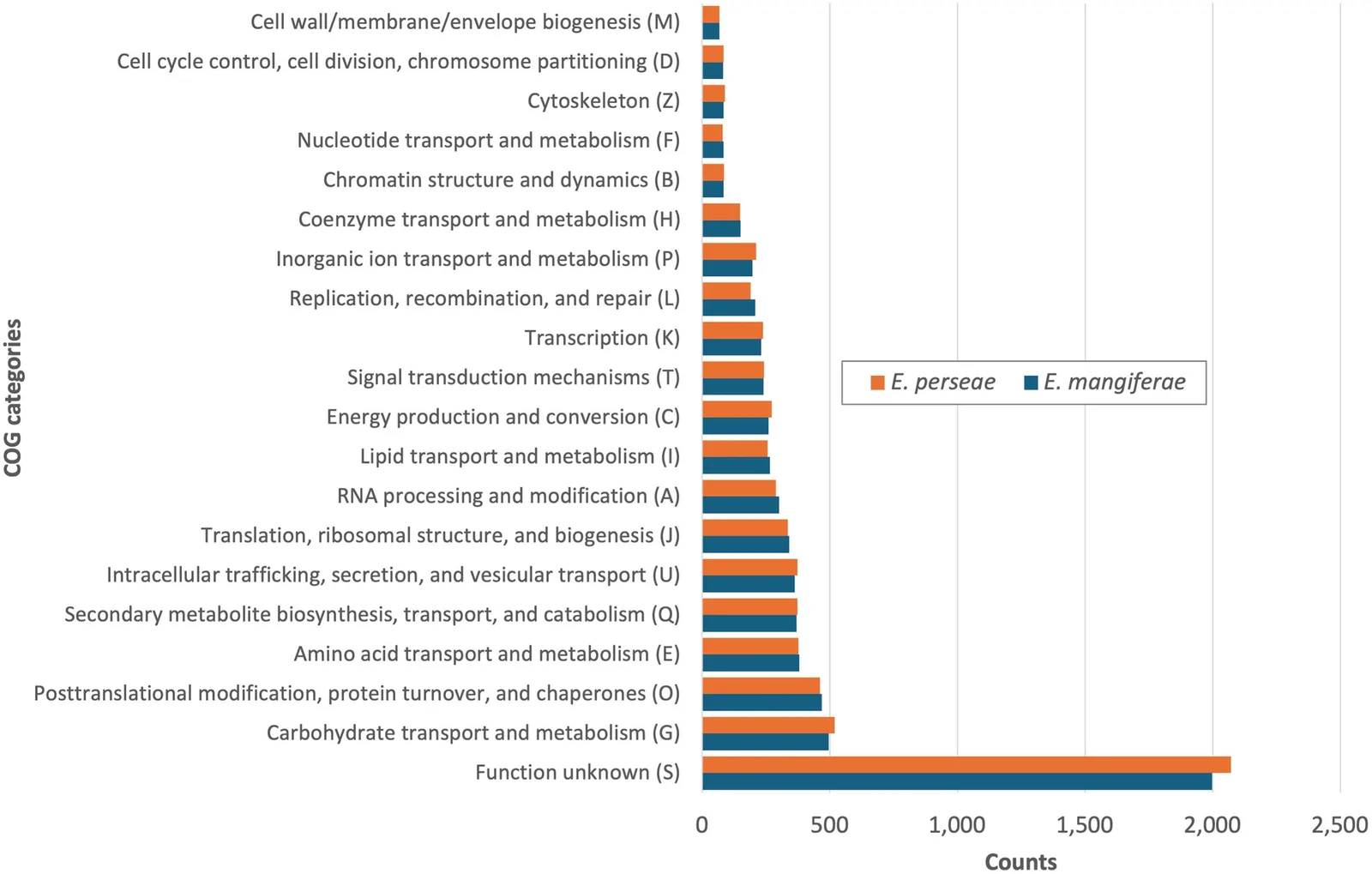
Distribution of Clusters of Orthologous Groups (COG) functional categories in the genomes of *Elsinoë mangiferae* CBS 226.50 and *E. perseae* CBS 406.34. Values/counts represent the number of predicted proteins assigned to each COG category based on EggNOG-mapper annotations. The classifications can be grouped into Information Storage and Processing (J, A, K, L, B), Cellular Processes and Signaling (D, T, M, Z, U, O), Metabolism (C, G, E, F, H, I, P, Q), or Poorly Characterized (S).

Independent functional annotation with eggNOG-mapper revealed that the majority of predicted proteins in both *E. mangiferae* and *E. perseae* were assigned to metabolism-related Clusters of Orthologous Groups (COGs) (Fig. 2, Supplementary Tables 7 and 8). Poorly characterized functions (e.g. unknown or general prediction) are also highly represented, especially in *E. mangiferae*. Information Storage and Processing, and Cellular Processes and Signaling COGs are present in moderate proportions, reflecting essential housekeeping functions. The most represented functional category was “Function Unknown” (S), accounting for 1,998 genes in *E. mangiferae* and 2,072 genes in *E. perseae* (Fig. 2, Supplementary Tables 7 and 8). This high proportion of uncharacterized genes suggests the presence of lineage-specific or poorly studied proteins, which may include novel factors involved in pathogenicity or adaptation to host environments (Plissonneau et al. 2016; Möller and Stukenbrock 2017; Rokas et al. 2018).

Among the well-characterized categories, several metabolic and regulatory functions were prominently represented (Fig. 2, Supplementary Tables 7 and 8). Carbohydrate transport and metabolism (G) and amino acid transport and metabolism (E) are highly represented, reflecting the importance of nutrient acquisition and processing during host colonization (Divon and Fluhr 2007; Ene et al. 2014; Tan and Oliver 2017). Posttranslational modification, protein turnover, and chaperones (O) were also abundant in both species, indicating a strong capacity for protein quality control and stress adaptation (Lo Presti et al. 2016; Ma et al. 2021; Zhao and Wang 2024). Additionally, both species encoded a substantial number of genes involved in intracellular trafficking, secretion, and vesicular transport (U). This category includes machinery for protein secretion, which may encompass effector proteins or enzymes involved in host manipulation (Giraldo et al. 2013; Lo Presti et al. 2016; John et al. 2021). Overall, the functional classification of predicted genes indicates that both *E. mangiferae* and *E. perseae* possess robust metabolic capabilities and cellular machinery essential for pathogenicity and survival in host environments. However, the high number of proteins with unknown functions highlights the need for further functional characterization.

#### KEGG pathway annotation

**Fig. 3.**
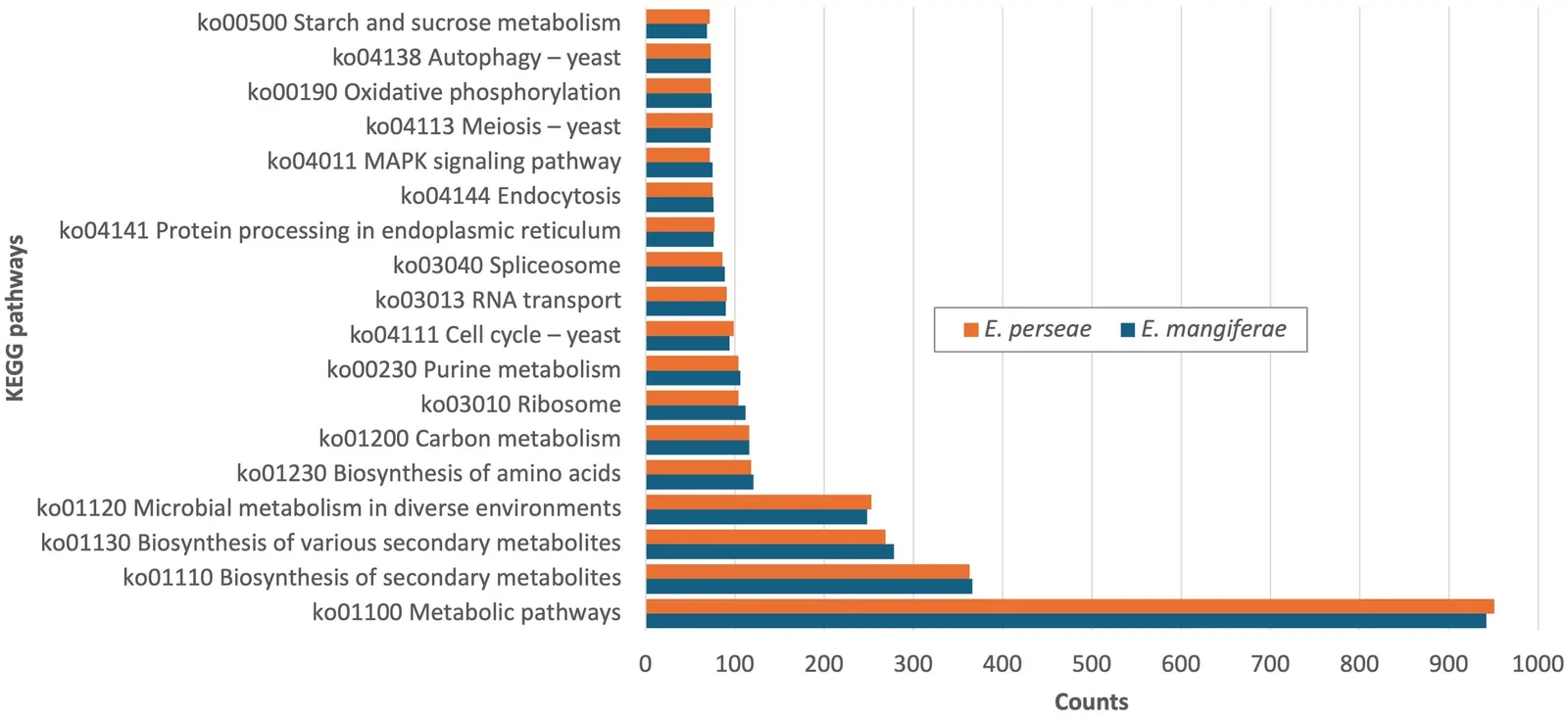
Top KEGG pathways identified in the genomes of *Elsinoë mangiferae* CBS 226.50 and *E. perseae* CBS 406.34 based on EggNOG-mapper annotations. Values/counts represent the number of predicted proteins assigned to each KEGG pathway. Because individual proteins may be associated with multiple pathways, counts are not mutually exclusive. Pathways are grouped into the major KEGG functional categories: Metabolism (ko01100, ko01110, ko01120, ko01130, ko01200, ko01230, ko00230, ko00500, ko00190), Genetic Information Processing (ko03010, ko03013, ko03040, ko03008), Cellular Processes (ko04111, ko04113, ko04138, ko04141, ko04144), and Environmental Information Processing (ko04011).

KEGG pathway analysis revealed that both *E. mangiferae* and *E. perseae* have similar patterns of abundance and diversity (Fig. 3, Supplementary Tables 7 and 8). They both encode complete pathways for primary and secondary metabolism, protein synthesis, and stress response. Interestingly, both genomes showed strong representation of pathways linked to secondary metabolite biosynthesis (ko01110, ko01130), oxidative stress tolerance (ko00190), and autophagy (ko04138), all of which are relevant to fungal pathogenicity (Juárez-Montiel et al. 2022; Yaakoub et al. 2022; Du et al. 2024; Hossain et al. 2025; Wu et al. 2025). For example, several *Elsinoë* species (e.g. *E. arachidis, E. australis*, and *E. fawcettii*) produce specialized toxins (e.g. elsinochromes) that are phototoxic and essential in pathogenesis (Liao and Chung 2008; Chung 2011; Jiao et al. 2019; Pham et al. 2025). The overall conserved KEGG pathways suggest shared strategies for host colonization and survival under plant immune pressure (Juárez-Montiel et al. 2022; Yaakoub et al. 2022; Du et al. 2024; Hossain et al. 2025; Wu et al. 2025). However, species-specific adaptations may lie in gene copy variation, divergence in secondary metabolite clusters, or regulatory differences, potentially explaining their host specialization (Gluck-Thaler and Slot 2018; Sánchez-Vallet et al. 2018).

#### Biosynthetic gene cluster diversity

**Fig. 4.**
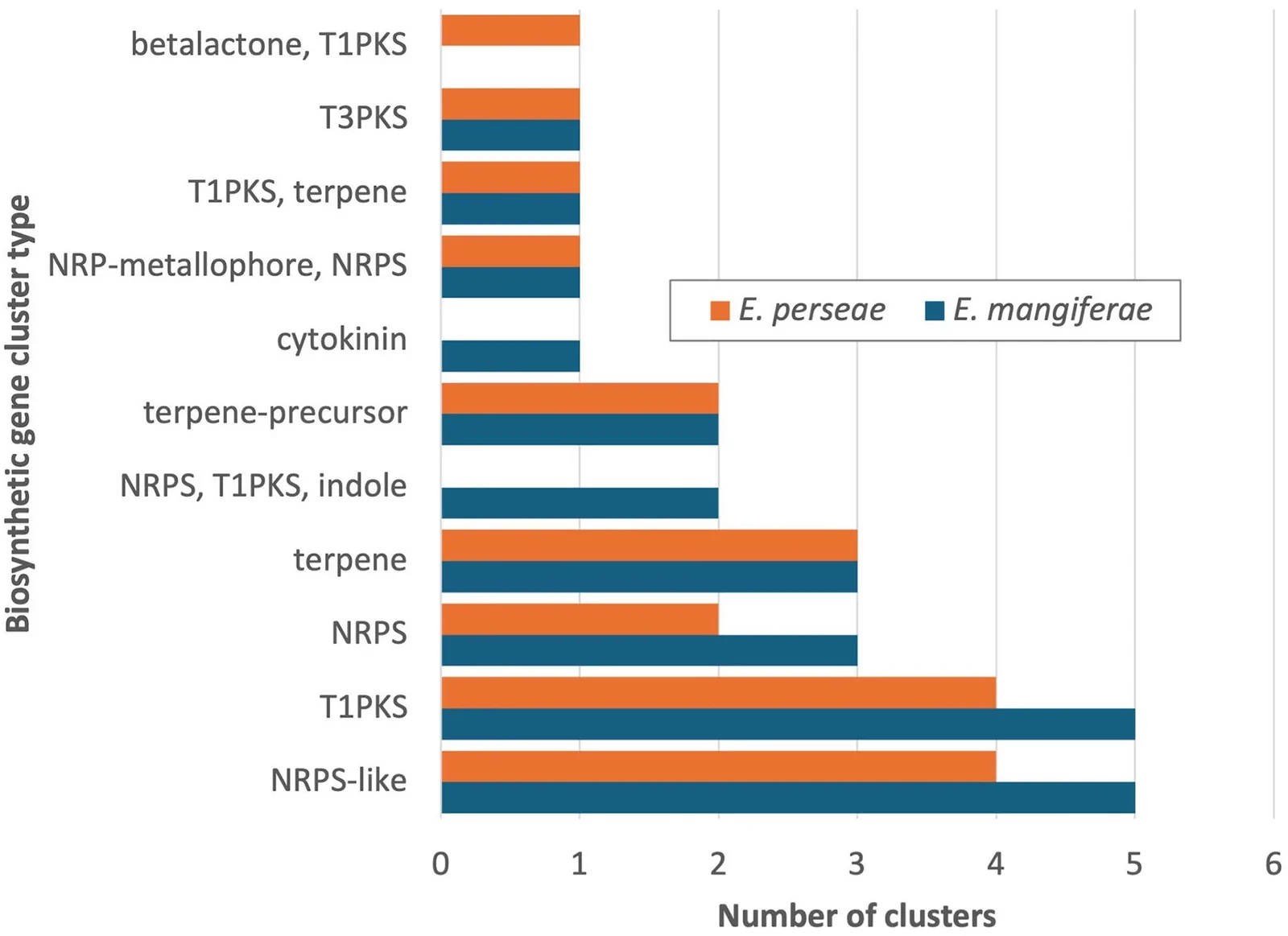
Biosynthetic gene clusters (BGCs) in *Elsinoë mangiferae* CBS 226.50 and *E. perseae* CBS 406.34 genomes. These are based on the results from antiSMASH. NRP, non-ribosomal peptide; NRPS, non-ribosomal peptide synthetase; PKS, polyketide synthases, Type 1 and 3.

AntiSMASH analyses identified several biosynthetic gene clusters (BGCs), i.e. 24 in *E. mangiferae* and 19 in *E. perseae* (Fig. 4, Supplementary Tables 9 and 10). Both species encoded core BGC types commonly associated with phytopathogenic fungi, including non-ribosomal peptide synthetases (NRPS), type I polyketide synthases (T1PKS), and terpene-related clusters (Kroken et al. 2003; Oide and Turgeon 2020; Yang et al. 2023). NRPS-like and T1PKS-like clusters were among the most abundant in both genomes. For example, both genomes had regions with moderate similarity scores (∼50–65%) to the NRPS Type I choline and the terpene clavaric acid (Supplementary Tables 9 and 10). Choline is an essential metabolite in the growth of filamentous fungi (Markham et al. 1993). Although the biological role of clavaric acid in fungi remains unclear, it has been identified as a compound with antitumor activity (Wang et al. 2021).

Only *E. mangiferae* contained BGCs showing similarity to the fusarin (NRPS-Type I PKS) and ACT-toxin II (PKS) clusters, although the similarity scores were moderate (∼50%). Fusarins are well-known mycotoxins (Wiemann et al. 2012). ACT-toxin II and similar metabolites have been reported in other plant pathogenic fungi (e.g. *Alternaria alternata* and *Fusarium* spp.), where they function as a host-selective toxin (HST) involved in pathogenicity and symptom development, and essential for virulence on susceptible hosts (Kohmoto et al. 1993; Takaoka et al. 2014; Elisyutikova et al. 2025; Ariantari et al. 2026). However, the observed similarity values are insufficient to infer production of these metabolites by *E. mangiferae*.

Additional BGCs with lower similarity scores included regions related to neosartorin, scytalone/T3HN, and elsinochrome C in both genomes (Supplementary Tables 9 and 10). Neosartorin is a secondary metabolite known for its antimicrobial activity (Ola et al. 2014), whereas scytalone and 1,3,8-trihydroxynaphthalene (T3HN) are key intermediates in the DHN-melanin biosynthesis pathway of many fungi (Chen et al. 2021; Verde-Yáñez et al. 2023). Elsinochrome is a photoactivated toxin that plays an important role in the pathogenicity of several *Elsinoë* species, as discussed above. Several additional BGCs could not be confidently identified, highlighting the need for further functional studies, expanded BGC reference databases, and the potential for the discovery of novel, lineage-specific secondary metabolites (Navarro-Muñoz et al. 2020; Kautsar et al. 2021; Blin et al. 2025).

#### Carbohydrate-active enzyme repertoires

**Fig. 5.**
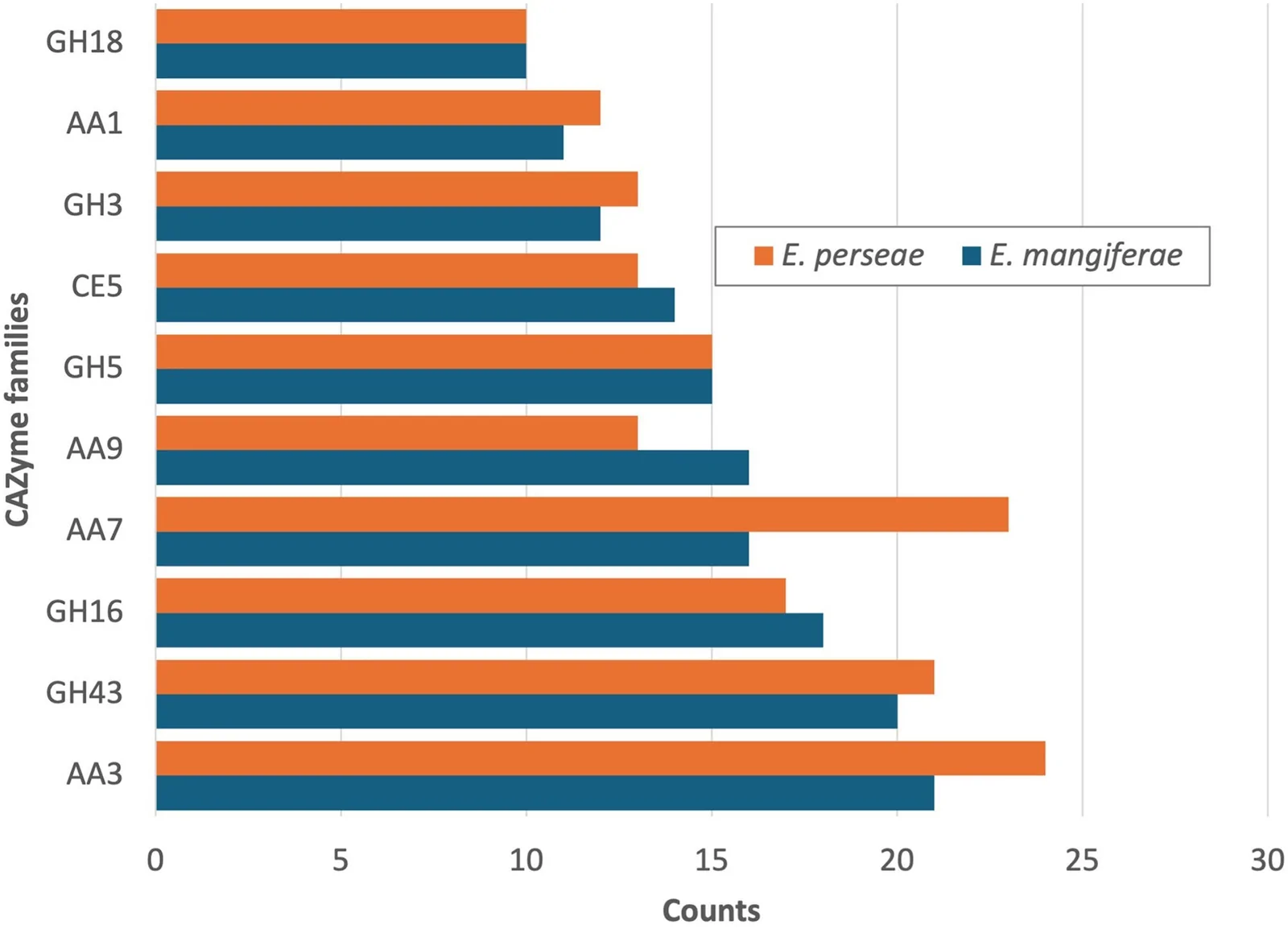
Top 10 most abundant carbohydrate-active enzyme (CAZyme) families identified in the genomes of *Elsinoë mangiferae* CBS 226.50 and *E. perseae* CBS 406.34 based on dbCAN annotations implemented through Funannotate. Values represent the number of predicted proteins assigned to each CAZyme family. Only the ten most abundant CAZyme families are shown; complete CAZyme annotations are provided in Supplementary Tables 5 and 6. AA, auxiliary activities; GH, glycoside hydrolases; CE, carbohydrate esterases. The families shown include oxidoreductases (AA3), hemicellulases (GH43), β-glucanases (GH16), oligosaccharide oxidases (AA7), lytic polysaccharide monooxygenases (AA9), cellulases/mannanases (GH5), cutinases/esterases (CE5), β-glucosidases (GH3), laccases (AA1), and chitinases (GH18).

Based on dbCAN annotations implemented through Funannotate, 411 and 443 carbohydrate-active enzymes (CAZymes) were identified in the genomes of *E. mangiferae* and *E. perseae*, respectively (Fig. 5; Supplementary Tables 5 and 6). Both species exhibited highly similar CAZyme repertoires, with Auxiliary Activities (AA), Glycoside Hydrolases (GH), and Carbohydrate Esterases (CE) among the most abundant classes. The most frequently represented families included AA3 and AA7 oxidoreductases, GH43 hemicellulose-degrading enzymes, GH16 β-glucan-modifying enzymes, AA9 lytic polysaccharide monooxygenases (LPMOs), GH5 cellulases/mannanases, and CE5 carbohydrate esterases. These enzymes are associated with the degradation or modification of plant-derived carbohydrates, including cellulose and hemicellulose, and may contribute to nutrient acquisition and host colonization (Brown et al. 2001; Lenardon et al. 2010; Rauwane et al. 2020; Jagadeeswaran et al. 2021; Takeda et al. 2022; Terrasan et al. 2022; Ota et al. 2023; Zhang et al. 2025). Particularly noteworthy was the presence of AA9, GH43, GH5, and CE5, all of which are involved in plant cell wall deconstruction (Jagadeeswaran et al. 2021; Takeda et al. 2022; Terrasan et al. 2022).

The overall CAZyme repertoires remain relatively modest compared with those reported for many broad-host-range necrotrophic fungi, consistent with the host-specific lifestyles of *Elsinoë* species (Koeck et al. 2011; Zhao et al. 2013; De Silva 2016; Li et al. 2021; Pham et al. 2025). These fungi are host-specific pathogens that likely employ stealth infection strategies (e.g. host immune evasion), relying less on extensive cell wall-degrading enzymes to avoid triggering plant immune responses (Van Der Does and Rep 2007; Zhao et al. 2013; Chellappan 2024). This is also common among hemibiotrophs, which often minimize their CAZy arsenal (Koeck et al. 2011; Zhao et al. 2013; De Silva 2016). Additionally, *Elsinoë* species may rely more on secondary metabolites and effectors rather than enzymatic degradation for host colonization (Van Der Does and Rep 2007; Chellappan 2024).

#### Additional functional annotations: secretome and candidate virulence factors

Funannotate identified 849 and 860 predicted secreted proteins in the genomes of *E. mangiferae* and *E. perseae*, respectively, based on SignalP annotations (Supplementary Tables 5 and 6). These proteins represent ∼9.3% and ∼10.8% of the predicted gene repertoires, indicating that both species possess substantial secretomes that may contribute to host colonization and pathogenicity. Secreted proteins play central roles in plant-pathogen interactions, including nutrient acquisition, degradation of host barriers, and modulation of host immune responses (Lo Presti et al. 2015; Toruño et al. 2016). In addition, MEROPS annotations identified 301 proteases in *E. mangiferae* and 307 in *E. perseae* (Supplementary Tables 5 and 6). Proteases are frequently associated with fungal virulence because they facilitate nutrient acquisition, degradation of host proteins, and suppression of plant defense responses (Xia 2004; Lee Erickson and Schuster 2024). Several secreted proteins identified through InterPro annotations are potentially relevant to pathogenicity. These included proteins containing Ecp2 effector-like domains (IPR029226), cell-surface Cu/Zn superoxide dismutases, and laccases/multicopper oxidases. Ecp2 was originally characterized as a secreted virulence-associated protein in fungal plant pathogens, and homologs have since been reported across diverse phytopathogenic fungi (Stergiopoulos et al. 2010; Rouxel and de Wit 2011; Lo Presti et al. 2015). Cell-surface Cu/Zn superoxide dismutases, found primarily in *E. perseae*, may help detoxify reactive oxygen species generated by the host during infection (Park and Son 2024). Laccases and multicopper oxidases, which were more abundant in *E. mangiferae*, have been associated with melanin biosynthesis, oxidative stress tolerance, and fungal virulence (Li et al. 2026). The large numbers of predicted secreted proteins and proteases, coupled with the presence of Ecp2-like proteins and oxidative stress-related enzymes, suggest that both species possess conserved repertoires of putative virulence factors involved in host colonization and disease development.

## Conclusions

We present draft genome assemblies for the ex-types of *Elsinoë mangiferae* (CBS 226.50) and *E. perseae* (CBS 406.34), 2 host-specific scab-causing pathogens of mango and avocado, respectively. Comparison of 5 assembly strategies demonstrated that ONT-only NextDenovo assemblies achieved the highest contiguity and overall assembly quality, outperforming both hybrid and alternative long-read approaches. The assemblies are 24.5 Mb (*E. mangiferae*) and 25.1 Mb (*E. perseae*) in size, with high contiguity (13 and 18 contigs, and N50 ∼2.2 and ∼1.9 Mb), and completeness (BUSCO scores 94.1 and 94.2%), providing valuable genomic resources for understudied *Elsinoë* species. These genomes are among the most contiguous and deeply sequenced *Elsinoë* genomes available to date. Functional annotation revealed conserved metabolic pathways, functionally relevant CAZyme repertoires, diverse secondary metabolite biosynthetic gene clusters, including clusters potentially associated with elsinochrome and ACT-toxin II production, and numerous predicted secreted proteins, proteases, and effector-like proteins that may contribute to pathogenicity. Both genomes also harbor a high proportion of uncharacterized genes, suggesting incomplete databases and/or the presence of lineage-specific genes. These assemblies and annotations establish a genomic foundation for future studies on pathogenicity, host specificity, and the evolution of scab disease in tropical and subtropical fruit crops.

## Supplementary Material

jkag177_Supplementary_Data

## Data Availability

Genome assemblies are publicly available in GenBank under the BioProject PRJNA1295512 (BioSamples SAMN50173967 and SAMN50173968). Supplementary Tables 1–10 and Supplementary Data (gene prediction gbk and gff3 files, and results from antiSMASH) are publicly available in Figshare (https://doi.org/10.6084/m9.figshare.c.8382724). The scripts used in the bioinformatics pipeline are publicly available in GitHub (https://github.com/EEscuderoL/Elsinoe-genomes-pipeline). Supplemental material available at G3 online.
