# Supplementary material for: Genomes of the ex-type strains of Elsinoë mangiferae and E. perseae, the causal agents of scab on mango and avocado
Source: G3 (Bethesda). 2026 Jul 2;16(9):jkag177. doi: 10.1093/g3journal/jkag177 (PMC13535502; doi:10.1093/g3journal/jkag177)
Supplement: Supplementary file 1 [file jkag177_supplementary_data.pdf]

Supplementary Information for Chaverri et al. Genomes of the ex-type strains of *Elsinoë mangiferae* and *E. perseae*, the causal agents of scab on mango and avocado (https://doi.org/10.6084/m9.figshare.c.8382724)

All these data and tables are deposited in FigShare. DOI numbers and links are provided.

Supplementary Table S1. List of *Elsinoë* genomes used in the phylogenomic analysis and Table 2. All assemblies, except those produced for this study (CBS 226.50 and CBS 406.34), were downloaded from GenBank. https://doi.org/10.6084/m9.figshare.29631002

Supplementary Table S2. Gene predictions for *E. mangiferae.*
https://doi.org/10.6084/m9.figshare.29631014

Supplementary Table S3. Gene predictions for *E. perseae.*
https://doi.org/10.6084/m9.figshare.29630999

Supplementary Table S4. Telomeric repeat summary in *E. mangiferae* and *E. perseae* genome assemblies. https://doi.org/10.6084/m9.figshare.29631008

Supplementary Table S5. Funannotate annotations for *E. mangiferae.*
https://doi.org/10.6084/m9.figshare.29630996

Supplementary Table S6. Funannotate annotations for *E. perseae.*
https://doi.org/10.6084/m9.figshare.29631056

Supplementary Table S7. Results of eggNOG-mapper for *E. mangiferae.*
https://doi.org/10.6084/m9.figshare.29631059

Supplementary Table S8. Results of eggNOG-mapper for *E. perseae.*
https://doi.org/10.6084/m9.figshare.32746887

Supplementary Table S9. Summary results of antiSMASH for *E. mangiferae.*
https://doi.org/10.6084/m9.figshare.32747082

Supplementary Table S10. Summary results of antiSMASH for *E. perseae.*
https://doi.org/10.6084/m9.figshare.32747097

Additional supplementary data:

Genome assemblies (fasta) and annotations (gbk and gff3) for CBS 406.34 and CBS 226.50.
https://doi.org/10.6084/m9.figshare.31855519

Raw antiSMASH results:
The ZIP files contain html, gbk, images, and all other results files from antiSMASH analysis. The file needs to be unzipped. Click on the html to visualize all results.
https://doi.org/10.6084/m9.figshare.29630990
https://doi.org/10.6084/m9.figshare.29630981
